# Evaluation of hybrid rye on growth performance, carcass traits, and efficiency of net energy utilization in finishing steers

**DOI:** 10.1093/tas/txaa173

**Published:** 2020-09-12

**Authors:** Warren C Rusche, Julie Ann Walker, Peter Sexton, Rebecca S Brattain, Zachary K Smith

**Affiliations:** 1 Department of Animal Science, South Dakota State University, Brookings, SD; 2 Department of Agronomy, Horticulture and Plant Science, South Dakota State University, Brookings, SD; 3 KWS Cereals USA, LLC, Champaign, IL

**Keywords:** feedlot, growth performance, rye, steers

## Abstract

Crossbred beef steers with a high percentage of Angus ancestry [*n* = 240, initial shrunk body weight (BW), 404 ± 18.5 kg] were used in a 117-d feedlot experiment to evaluate the effect of hybrid rye (Rye; KWS Cereals USA, LLC, Champaign, IL) as a replacement for dry-rolled corn (DRC) on growth performance, carcass traits, and comparative net energy (NE) value in diets fed to finishing steers. Rye from a single hybrid (KWS Bono) with an ergot alkaloid concentration of 392 ppb was processed with a roller mill to a processing index (PI) of 78.8 ± 2.29. Four treatments were used in a completely randomized design (*n* = 6 pens/treatment; 10 steers/pen), where DRC (PI = 86.9 ± 4.19) was replaced by varying proportions of Rye [DRC:Rye, dry matter (DM) basis (60:0), (40:20), (20:40), and (0:60)]. Liver abscess scores and carcass characteristics were collected at the abattoir. Carcass-adjusted performance was calculated from hot carcass weight (HCW)/0.625. Performance-adjusted NE was calculated using carcass-adjusted average daily gain (ADG), DM intake (DMI), and mean equivalent shrunk BW with the comparative NE values for rye calculated using the replacement technique. Data were analyzed using the GLIMMIX procedure of SAS 9.4 (SAS Inst. Inc., Cary, NC) with pen as the experimental unit. Treatment effects were tested using linear and quadratic contrasts, as well as between diets with and without Rye. Replacing DRC with Rye linearly decreased (*P* ≤ 0.01) carcass-adjusted final BW, ADG, DMI, and gain:feed (G:F). Feeding rye linearly decreased HCW and longissimus muscle area (*P* ≤ 0.04). Distributions of liver scores and USDA grades for quality and yield were unaffected by treatment (*P* ≥ 0.09). Estimated replacement NE for maintenance (NEm) and gain (NEg) values for rye, when included at 60% of diet DM, were 1.90 and 1.25 Mcal/kg, respectively. Rye can be a suitable feed ingredient in finishing diets for feedlot steers. Estimated replacement values of Rye when fed at 60% of diet DM closely agreed with current tabular standards but, when included at 20% of diet DM, estimated NEm and NEg values of Rye were increased 9.5% and 12.8%, respectively. Net energy value of Rye for gain is approximately 84% compared to DRC; thus, the complete replacement of DRC with Rye depressed DMI, ADG, G:F, and carcass weight.

## INTRODUCTION

Increasing crop-rotation diversity offers a number of benefits to an integrated crops–livestock production system, including greater yield resiliency and enhanced yield increases compared to a monoculture or two-crop rotation (Bowles et al., 2020). Diversified crop rotations, when combined with livestock production, also reduce month-to-month variation in labor requirements compared to a corn–soybean rotation with livestock (Poffenbarger et al., 2017).

Cereal rye offers several attributes that warrant consideration for inclusion as a component of an integrated crops–livestock system. Rye can be grazed, harvested for forage, or allowed to reach maturity for the harvest of grain and straw. Furthermore, rye is harvested earlier than row crops, allowing for greater manure application flexibility or planting of short-season forage crops if conditions allow. Newer hybrid rye (Rye) germplasms are particularly promising because of their enhanced yield potential and decreased ergot incidence compared to traditional open-pollinated rye cultivars (Hansen et al., 2004).

Multiple potential uses enhance the utility and acceptance of any crop by providing additional options and lessening the reliance on any one market channel. Cereal rye grain has not been traditionally thought of as a suitable cereal grain for finishing cattle. It has been recommended to only feed cereal rye to finishing cattle in limited amounts because of the negative effects of ergot ingestion and observed decreases in dry matter intake (DMI; Matsushima, 1979). Those recommendations were made before the introduction of novel Rye germplasm with decreased ergot risk and before the widespread adoption of corn-processing coproducts in finishing diets.

The objectives of this experiment were to determine the effects of Rye inclusion on DMI, growth performance, and feed efficiency in finishing beef steers and to estimate net energy (NE) value. Our hypothesis was that cereal rye could be substituted for dry-rolled corn (DRC) in finishing beef diets and that increased inclusion rates would decrease growth performance and feed efficiency with no negative effects on carcass characteristics or the severity and incidence of liver abscesses.

## MATERIALS AND METHODS

All procedures involving the use of animals in this experiment were approved by the South Dakota State University Institutional Animal Care and Use Committee (approval number 19-047E). The experiment was conducted at the South Dakota State University Southeast Research Farm (SERF) located near Beresford, SD.

### Experimental Design and Treatments

Four treatments were used in a completely randomized design to evaluate animal performance and carcass traits and to estimate the NE value for Rye. Hybrid rye was substituted for DRC as follows: a basal finishing diet formulated [dry matter (DM) basis] with 60% corn grain (DRC:Rye, 60:0) and three additional diets formulated with increasing proportions of Rye (40:20, 20:40, and 0:60). All rye grain used was from the same hybrid (KWS Bono, KWS Cereals, LLC; Champaign, IL) and from a single source. Each truckload of Rye was sampled on arrival at SERF and composited for ergot alkaloid analysis. Total ergot alkaloid concentration from the composited sample was 392 ppb on a DM basis (Table 1; NDSU Veterinary Diagnostic Laboratory, Fargo, ND) and less than the recommended maximum ergot alkaloid concentration of 2 ppm for cattle diets (Coufal-Majewski et al., 2016).

**Table 1.** Hybrid cereal rye ergot alkaloid concentration (DM basis)^*a*,*b*^

Ergot alkaloid	Concentration, ppb
Ergosine	70
Ergotamine	25
Ergocornine	31
Ergocryptine	138
Ergocristine	47
Ergosinine	28
Ergotaminine	<20
Ergocorinine	<20
Ergocryptinine	32
Ergocristinine	21
Total	392

^*a*^North Dakota State University Veterinary Diagnostic Laboratory.

^*b*^Detection limit = 20 ppb.

### Animals, Initial Processing, and Study Initiation

Crossbred beef steers with a high percentage of Angus ancestry [*n* = 240, initial shrunk body weight (BW), 404 ± 18.5 kg] were used in this experiment. Steers were sourced from a single consignment at one South Dakota auction facility and delivered to SERF. Steers were fed in 24 dirt-surfaced pens, resulting in six replications per treatment and 60 steers per treatment (*n* = 10 steers/pen). The dirt-surfaced pens had a 6.1-m concrete bunk apron, a continuous flow waterer on the fence line located 0.6 m from the bunk apron, and provided 54.4 m^2^ of pen space per steer and 61 cm of linear bunk space per steer. Cattle were processed on September 6, 2019, where BW was collected to be used for allotment purposes, a unique identification tag was applied to each steer, vaccines were administered against respiratory pathogens: infectious bovine rhinotracheitis, bovine viral diarrhea types 1 and 2, parainfluenza-3 virus, and bovine respiratory syncytial virus (Bovi-Shield Gold 5, Zoetis, Parsippany, NJ) and clostridial species (Ultrabac 7/Somubac, Zoetis), and administered pour-on moxidectin (Cydectin, Bayer, Shawnee Mission, KS). The experiment was initiated on September 10, 2019 with a 19-d adaptation period and a 98-d finishing period, resulting in a total experiment length of 117 d. On September 30, 2019 (day 19), steers were administered a steroidal implant (200 mg trenbolone acetate and 28 mg estradiol benzoate; Synovex Plus, Zoetis).

### Diets and Intake Management

Steers were fed once daily. Steers were stepped up to the final diet over a 19-d period. From day 8 to day 14, Rye was introduced to the step-up diets at 40% of the ultimate inclusion rate (0%, 8%, 16%, and 24%, respectively) with the final proportions of Rye fed in experimental diets from day 15 to day 19. The final diets fed (days 20–117) are presented in Table 2. Bunks were managed so as to be devoid of feed at 0800 h. Feed intake and diet formulations were summarized at weekly intervals. Steers that were removed from the study or that died during the study were assumed to have consumed feed equal to the pen mean DMI up to the point of removal or death. Two steers (one from 60:0 and one from 40:20) died or were removed from the study for reasons unrelated to dietary treatment; thus, all data are reported on a deads and removals excluded basis.

**Table 2.** Composition of experimental finishing diets fed from day 19 to day 117 (DM basis)

Item	DRC: rye grain inclusion (DM basis)			
	60:0	40:20	20:40	0:60
Ingredient composition, %				
DRC	60.34	40.33	20.22	0.00
Hybrid rye	0.00	19.91	39.93	60.04
MDGS^*a*^	18.90	18.95	19.00	19.05
Corn silage	16.84	16.89	16.93	16.97
Liquid supplement^*b*^	3.91	3.92	3.93	3.94
Nutrient composition^*c*^				
NEm, Mcal/kg	2.08	2.02	1.95	1.89
NEg, Mcal/kg	1.41	1.35	1.30	1.25
CP, %	12.78	13.62	14.47	15.32
NDF, %	18.90	20.91	22.94	24.98
ADF, %	9.88	11.10	12.32	13.54
Ash, %	4.83	4.92	5.01	5.09
EE, %	4.69	4.35	4.01	3.67

^*a*^MDGS, modified distiller’s grains plus solubles.

^*b*^Provided 30 g/907 kg of monensin, as well as vitamins and minerals to exceed requirements (NASEM, 2016).

^*c*^Tabular NE from (Preston, 2016) and actual nutrient compositions from weekly assays of the ingredients.

**Figure 1.**
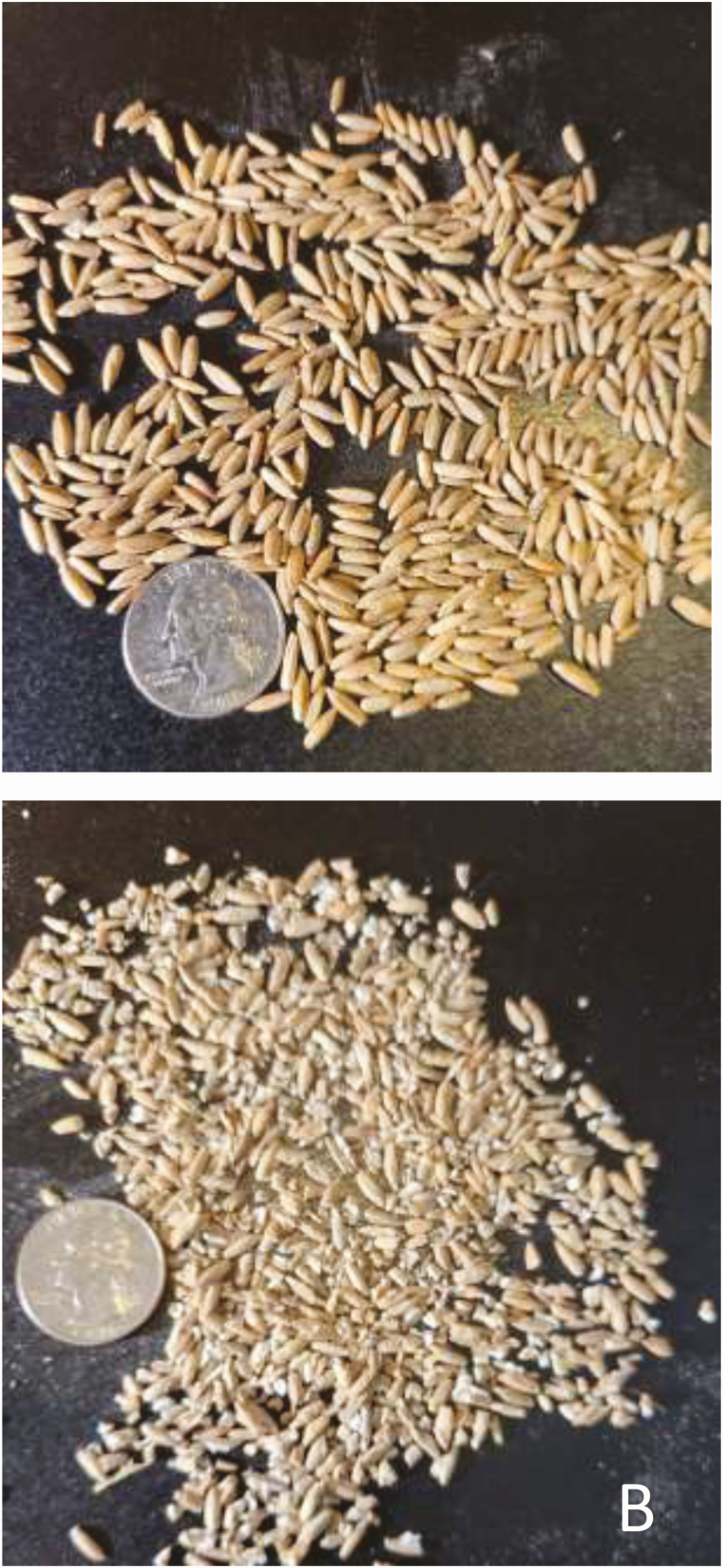
Depicts unprocessed whole rye (A) and processed rye (B), PI = 78.8 ± 2.29. PI is defined as: (g/L processed grain/g/L unprocessed grain) × 100.

Rye was processed by passing whole rye through a roller mill (Lone Star Enterprises, Lennox, SD). The rolls were 23 × 30 cm with 4.7 corrugations per centimeter in a round bottom v pattern and ran at 857.5 rpm at a 1:1 ratio. Corrugations in one roller were straight, while the second roller was machined with a 12.7-cm spiral design. Rolls were adjusted so that the processing index (PI) for Rye was 78.8 ± 2.29 as described by (Yang et al., 2014), where PI was defined as the volume weight (grams per liter) of the grain (as is) after processing expressed as a percentage of the volume weight before processing. Rye samples (processed and unprocessed) were analyzed for particle size distribution and geometric mean diameter at Ward Laboratories in Kearney, NE (Table 3). Samples were split using a riffle splitter, and a 100-g subsample was weighed and sieved through a set of eight circular sieves (3,350 μm; 1,700 μm; 1,180 μm; 850 μm; 600 μm; 425 μm; 212 μm; 53 μm, and pan) using a sieve shaker for 10 min. After the sample was shaken, the weight of the material on each sieve was recorded. No agitators or dispersion agents were used in the analysis. Representative visual examples of the degree of processing compared to whole rye are presented in Fig. 1. Dry-rolled corn was processed similarly with a PI of 86.9 ± 4.19. Ingredient samples were collected weekly and DM calculated after drying in a forced-air oven at 60 °C until no further weight change occurred. Weekly DM values for each ingredient were used to calculate DMI and actual DM ingredient inclusions. Weekly ingredient samples were stored in a freezer at −20 °C until nutrient analyses were completed. After DM determination (method no. 935.29; AOAC, 2012), weekly samples from each ingredient were analyzed for N (method no. 968.06; AOAC, 2016; Rapid Max N Exceed; Elementar; Mt. Laurel, NJ) and ash (method no. 942.05; AOAC, 2012). Modified distillers grains samples were analyzed for ether extract content using an Ankom Fat Extractor (XT10; Ankom Technology, Macedon, NY). Percentages of acid detergent fiber (ADF) and neutral detergent fiber (NDF) were assumed to be 3% and 9% for corn and 9% and 19% for Rye, respectively (Preston, 2016). Analysis of ADF and NDF composition for all other feeds was conducted as described by Van Soest et al. (1991). Nutrient composition values for Rye and DRC are presented in Table 4. Dietary metabolizable protein supply and balance were determined post hoc using the Beef Cattle Nutrient Requirements Model (NASEM, 2016) using observed performance variables; solution type was set at empirical calculations.

**Table 3.** Whole and processed rye particle size distribution, geometric mean diameter (GMD), and geometric mean diameter SD (GMDSD)

Item	Whole rye (%, retained)	Processed rye (%, retained)
Screen size, μm		
3,350	0.0	0.0
1,700	96.1	78.1
1,180	3.9	17.8
850	0.0	2.7
600	0.0	0.6
425	0.0	0.3
212	0.0	0.4
53	0.0	0.1
Pan	0.0	0.0
GMD, μm	2,339	2,081
GMDSD	1.1	1.4

**Table 4.** Nutrient composition of Rye and DRC (DM basis)

Item	Rye (*n* = 17)	DRC (*n* = 17)
Nutrient composition, %		
DM (as-is basis)	88.83 ± 0.962	88.00 ± 2.149
Crude protein	11.58 ± 0.464	7.32 ± 0.321
Ash	2.00 ± 0.071	1.53 ± 0.168
PI^*a*^	78.8 ± 2.29	86.9 ± 4.19

^*a*^PI = (g/L processed grain/g/L unprocessed grain) × 100.

### Cattle Management and Data Collection

Steer BW were recorded at the time of study initiation, days 19, 47, and 75, and the morning of study termination on day 117 for the calculation of growth performance. Body weights were measured before the morning feeding with a 4% pencil shrink applied to the initial and final BWs. Wet weather combined with temperatures generally greater than 0 °C during the final 40 d of this experiment resulted in greater than normal amounts of mud at harvest. Therefore, carcass-adjusted performance using hot carcass weight (HCW) adjusted to a common dressing percentage (DP) of 62.5% was used to determine cumulative performance and efficiency measures with unshrunk BW used for interim performance measures.

Cattle were weighed off test when they were visually appraised to have 1.27 cm of fat at the 12th rib. Cattle were shipped 48 h after final BW determination and harvested the next day at Tyson Fresh Meats in Dakota City, NE. Steers were commingled at the time of study termination and remained as such until 0700 h the morning after shipping. Prevalence of abscessed livers and abscess severity were determined by a trained technician using the Elanco system as Normal (no abscesses), A− (one or two small abscesses or abscess scars), A (two to four well-organized abscesses less than 2.5 cm diameter), or A+ (one or more large active abscesses greater than 2.5 cm diameter with inflammation of surrounding tissue). Video image data were obtained from the plant for longissimus muscle area, rib fat (RF), calculated USDA Yield Grade (YG), and USDA marbling scores. The DP was calculated as HCW/(final BW × 0.96). Estimated empty body fat (EBF) percentage and final BW (AFBW) at 28% EBF were calculated from observed carcass traits (Guiroy et al., 2002) and the proportion of closely trimmed boneless retail cuts from carcass round, loin, rib, and chuck (Retail Yield, RY; Murphey et al., 1960).

Performance-adjusted NE (paNE) was calculated from daily energy gain (EG; Mcal/d): EG = [carcass-adjusted average daily gain (ADG) from d 20 to 117]^1.097^ × 0.0557W^0.75^, where W is the mean equivalent shrunk BW [shrunk BW × (478/AFBW), kg; (NRC, 1996)] for the period from day 20 to day 117. Maintenance energy required (EM; Mcal/d) was calculated by the following equation: EM = 0.077BW^0.75^ (Lofgreen and Garrett, 1968), where BW is the mean shrunk BW (using the average of carcass-adjusted final BW and BW from day 20). Using the estimates required for maintenance and gain, the paNE for maintanence (paNEm) and gain (paNEg) values (Owens and Hicks, 2019) of the diet were generated using the quadratic formula: $x=\;\frac{-b\pm \sqrt{b^{2}-4ac}}{2c}$, where *x* = NEm, Mcal/kg, *a* = −0.41 EM, *b* = 0.877 EM + 0.41 DMI + EG, *c* = −0.877 DMI, and NEg was determined from: 0.877 NEm – 0.41 (Zinn and Shen, 1998; Zinn et al., 2008).

The comparative NEm values for Rye were estimated using the replacement technique. Given that the NEm value of DRC was 2.17 Mcal/kg (NASEM, 2016), the comparative NEm values for Rye were estimated as follows (Estrada-Angulo et al., 2019): Rye NEm (Mcal/kg) = [(test diet paNEm − control diet paNEm)/RYE_*y*_] + 2.17, where RYE_*y*_ represents the inclusion of Rye that replaced DRC in the diet (0.1991, 0.3993, and 0.6004), respectively. The same was done for NEg, assuming DRC had an NEg value (Mcal/kg) of 1.49 (NASEM, 2016).

### Statistical Analysis

Growth performance, carcass traits, and efficiency of dietary energy utilization were analyzed as a completely randomized design using the MIXED procedure of SAS 9.4 (SAS Inst. Inc., Cary, NC) with pen as the experimental unit. The model included the fixed effect of dietary treatment. Least squares means were generated using the LSMEANS statement of SAS and treatment effects were evaluated using orthogonal polynomials (Steel and Torrie, 1960). Dry matter intake was evaluated in the MIXED procedure of SAS 9.4 using repeated measures; the model included the fixed effects of treatment, day, and their interaction; day was included as the repeated variable; and pen was considered the experimental unit. The covariance structure with the lowest Akaike information criterion was used. Distribution of USDA YG and Quality Grade (QG) and liver abscess severity and prevalence data were analyzed as binomial proportions in the GLIMMIX procedure of SAS 9.4 with fixed effects in the model as described previously. An α of 0.05 or less determined significance, and tendencies are discussed between 0.05 and 0.10.

## RESULTS

Rye improved performance with linear increases in BW, ADG, DMI, and gain:feed (G:F) (*P* = 0.01; Table 5) during the initial 19-d adaptation period (with Rye fed from day 8 to day 19). Some positive responses to Rye were maintained during the day 20 to day 47 period, with a quadratic increase in BW (*P* = 0.03) and a tendency for increased ADG and G:F (*P* ≤ 0.07). Increased Rye linearly decreased ADG (*P* = 0.01) and quadratically decreased BW (*P* ≤ 0.04) from day 48 to day 117. Dry matter intake decreased quadratically (*P* = 0.03) from day 48 to day 75 with reduced DMI for 0:60 compared to treatments with lesser inclusion of Rye. Dry matter intake linearly decreased (*P* = 0.01) with increasing Rye inclusion from day 76 to day 117. Feed efficiency was linearly decreased by increased Rye (*P =* 0.05) from day 48 to day 75 but unaffected by treatment from day 76 to trial termination.

**Table 5.** Influence of replacing DRC with Rye grain on interim period steer growth performance

Item	DRC:Rye grain inclusion, % DM basis				SEM	*P*-value		
	60:0	40:20	20:40	0:60		0 vs. Rye	L	Q
Allotment BW, kg^*a*,*b*^	429	429	429	429	—	—	—	—
Initial BW, kg^*c*^	418	420	421	423	—	—	—	—
Initial to day 19								
BW day 19, kg	445	450	455	459	2.4	0.01	0.01	0.84
ADG, kg	1.40	1.54	1.78	1.90	0.121	0.02	0.01	0.94
DMI, kg	9.48	9.50	9.51	9.52	0.005	0.01	0.01	0.27
G:F	0.139	0.159	0.182	0.196	0.0128	0.02	0.01	0.66
Days 20–47								
BW day 47, kg	524	532	536	534	2.2	0.01	0.01	0.03
ADG, kg	2.84	2.93	2.90	2.66	0.087	0.87	0.13	0.07
DMI, kg	11.05	11.05	11.04	11.05	0.001	0.01	0.01	0.01
G:F	0.256	0.265	0.261	0.239	0.0078	0.83	0.10	0.06
Days 48–75								
BW day 75, kg	612	620	619	615	2.5	0.07	0.62	0.02
ADG, kg	3.14	3.13	2.97	2.88	0.064	0.07	0.01	0.51
DMI, kg	13.65	13.60	13.43	13.15	0.050	0.01	0.01	0.03
G:F	0.230	0.230	0.221	0.218	0.0046	0.23	0.05	0.78
Days 76–117								
BW day 117, kg	704	713	704	689	5.4	0.83	0.05	0.04
ADG, kg	2.17	2.21	2.02	1.79	0.107	0.20	0.01	0.21
DMI, kg	14.63	14.27	13.84	13.33	0.184	0.01	0.01	0.68
G:F	0.145	0.155	0.145	0.133	0.0065	0.91	0.14	0.11

^*a*^No shrink was applied to any BW measures.

^*b*^BW collected on September 6, 2020.

^*c*^Cattle were allotted using BW from September 6, 2019; BW from September 10, 2019 was used as initial on-test BW.

**Figure 2.**
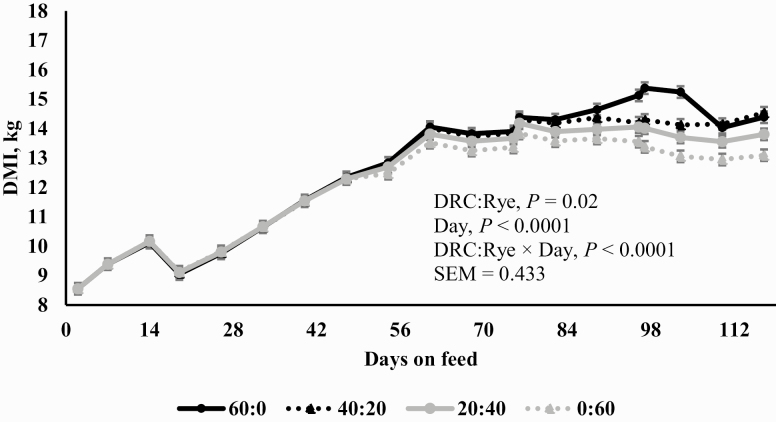
Effect of treatment on DMI over the experimental period. Hybrid rye (Rye) was substituted for DRC as follows: a basal finishing diet formulated (DM basis) 60% corn grain (DRC:Rye, 60:0) and three additional diets formulated with increasing proportions of Rye (40:20, 20:40, and 0:60). For each of the four treatments, there were 60 steers housed in six pen. All rye grains used were from the same hybrid (KWS Bono, KWS Cereals, LLC, Champaign, IL) and from a single source. The experiment was analyzed as a completely randomized design using repeated measures.

Using carcass-adjusted cumulative performance, Rye inclusion linearly decreased carcass-adjusted final BW, ADG, and G:F (*P* = 0.01; Table 6). There was an interaction (*P* < 0.0001) between DRC:Rye and days on feed, where DMI did not differ between treatments initially but diverged during the experiment, resulting in decreased DMI with increased Rye inclusion (*P* = 0.02; Fig. 2). Using the period from when steers were on the final diet (days 20–117; the energetics assessment period), rye inclusion linearly decreased paNE values (*P* = 0.01; Table 6) with no effect on the observed/expected NE (*P* ≥ 0.31). Comparative NEm and NEg values for Rye at 20%, 40%, and 60% inclusion levels were 2.08 and 1.41, 1.93 and 1.28, and 1.90 and 1.25 Mcal/kg for maintenance and gain, respectively.

**Table 6.** Effect of replacing DRC with Rye grain on carcass-adjusted growth performance of feedlot steers and dietary energy

Item	DRC:Rye grain inclusion, % DM basis				SEM^*b*^	*P*-value^*a*^		
	60:0	40:20	20:40	0:60		0 vs. Rye	L	Q
Initial BW, kg^*c*^	401	404	405	406	—	—	—	—
Final BW, kg^*d*^	650	648	632	620	4.9	0.01	0.01	0.32
ADG, kg	2.12	2.09	1.94	1.83	0.030	0.01	0.01	0.36
DMI, kg	12.71	12.57	12.38	12.13	0.067	0.01	0.01	0.42
G:F	0.167	0.166	0.157	0.150	0.0030	0.02	0.01	0.38
Energetics assessment period (days 20–117)								
d 19 BW, kg^*c*^	427	432	437	441	2.3	0.01	0.01	0.84
Final BW, kg^*d*^	650	648	632	620	4.9	0.01	0.01	0.32
ADG, kg	2.27	2.20	1.99	1.83	0.053	0.01	0.01	0.40
DMI, kg	13.34	13.16	12.93	12.64	0.080	0.01	0.01	0.43
G:F	0.170	0.167	0.154	0.145	0.0034	0.01	0.01	0.39
MP balance^*e*^, g/d	590	544	454	363	—	—	—	—
paNE, Mcal/kg^*f*^								
Maintenance	2.07	2.05	1.98	1.91	0.027	0.01	0.01	0.34
Gain	1.41	1.39	1.32	1.26	0.024	0.01	0.01	0.34
Observed/expected dietary NE^*g*^								
Maintenance	0.99	1.02	1.01	1.01	0.014	0.31	0.65	0.36
Gain	1.00	1.03	1.02	1.01	0.018	0.35	0.73	0.35
Estimated NE value of Rye, Mcal/kg^*h*^								
Maintenance	—	2.08	1.93	1.90	—	—	—	—
Gain	—	1.41	1.28	1.25	—	—	—	—

^*a*^0 vs. Rye = 60:0 vs. 40:20, 20:40, 0:60; L = Linear; Q = Quadratic.

^*b*^Pooled SEM.

^*c*^BW was shrunk 4% to account for digestive tract fill.

^*d*^Carcass adjusted using HCW/0.625.

^*e*^Daily metabolizable protein balance determined using the NASEM (2016) Beef Cattle Nutrient Requirements Model.

^*f*^ Owens and Hicks 2019.

^*g*^paNE/tabular trial NE.

^*h*^Net energy values for Rye derived using the replacement technique, assuming that NEm and NEg of DRC are 2.17 and 1.49 Mcal/kg, respectively (NASEM, 2016).

Hot carcass weight linearly decreased with increased Rye (*P* = 0.01; Table 7). The DP decreased quadratically with increased Rye inclusion (*P* = 0.02). Rib fat, kidney, pelvic, and heart fat (KPH) percentage, YG, RY, and estimated EBF percentage were unaffected by treatment (*P* ≥ 0.14). Dietary treatment tended (*P* = 0.07) to affect marbling quadratically with reduced marbling in the 0:60 treatment compared to treatments with lesser inclusions of Rye. Longissimus muscle area decreased with increased Rye inclusion (*P* = 0.04), as did AFBW (*P* = 0.01). Dietary treatment did not affect distributions of USDA YG or QG or severity or prevalence of liver abscess scores (*P* ≥ 0.09).

**Table 7.** Effect of replacing DRC with Rye grain on carcass traits and abscessed liver prevalence in feedlot steers

DRC:Rye grain inclusion, % DM basis					SEM^*b*^	*P*-value^*a*^		
	60:0	40:20	20:40	0:60		0 v Rye	L	Q
HCW, kg	406	405	395	388	3.0	0.01	0.01	0.33
DP, %^*c*^	60.10	59.12	58.42	58.56	0.221	0.01	0.01	0.02
RF, cm	1.30	1.30	1.30	1.24	0.036	0.78	0.46	0.55
Longissimus muscle area, cm^2^	83.3	84.6	82.1	80.8	1.00	0.52	0.04	0.22
Marbling^*d*^	474	478	485	445	11.3	0.74	0.14	0.07
KPH, %	1.79	1.80	1.81	1.79	0.014	0.59	0.71	0.48
YG	3.40	3.32	3.37	3.32	0.063	0.43	0.54	0.85
RY, %^*e*^	49.67	49.83	49.72	49.82	0.136	0.46	0.60	0.82
Estimated EBF, %	30.29	30.19	30.43	29.78	0.253	0.59	0.27	0.29
Final BW at 28% EBF (AFBW), kg	599	599	581	580	4.5	0.02	0.01	0.99
YG distribution						*P*-value		
YG 1, %	1.70	0.00	0.00	0.00	0.833	0.41		
YG 2, %	13.70	23.89	11.67	21.67	5.261	0.31		
YG 3, %	64.26	64.26	78.33	70.00	8.218	0.59		
YG 4, %	20.37	11.85	10.00	8.33	5.453	0.43		
QG distribution								
Prime, %	0.00	0.00	3.33	0.00	1.054	0.09		
Premium choice, %^*f*^	29.07	34.07	30.00	21.67	6.517	0.60		
Choice, %	50.37	50.93	53.34	48.33	7.590	0.97		
Select, %	20.56	15.00	13.33	30.00	4.966	0.11		
Abscessed liver scores^*g*^								
Normal, %	69.44	74.63	65.00	70.00	4.909	0.60		
A^−^, %	13.52	5.00	13.33	13.33	4.419	0.46		
A, %	8.52	10.00	6.67	6.67	3.360	0.87		
A^+^, %	8.52	10.37	15.00	10.00	4.365	0.75		

^*a*^0 vs. Rye = 60:0 vs. 40:20, 20:40, 0:60; L = Linear; Q = Quadratic.

^*b*^Pooled SEM.

^*c*^HCW/final BW shrunk 3%.

^*d*^USDA marbling score 400 = small^0^ = low choice; 500 = modest^0^ = average choice.

^*e*^As a percentage of HCW.

^*f*^Average or high-choice QG.

^*g*^Normal (no abscesses), A^−^ (one or two small abscesses or abscess scars), A (two to four well-organized abscesses <2.5 cm diameter), and A^+^ (one or more large active abscesses >2.5 cm diameter with inflammation of surrounding tissue).

## DISCUSSION

Estimated NEm and NEg values for Rye when included at 60% of diet DM (1.90 and 1.25 Mcal/kg, respectively) in the current experiment agree closely with previously published values for rye grain (1.97 and 1.32 Mcal/kg; NASEM, 2016; 1.90 and 1.23 Mcal/kg, Preston, 2016). Rye included at 20% of diet DM blended with DRC resulted in NEm and NEg estimates 9.5% and 12.8% greater when compared to Rye fed at 60%, consistent with positive associative effects observed when blends of wheat and corn were fed (Kreikemeier et al., 1987). Therefore, the combination of DRC and Rye could be a tool to optimize the NE content of Rye when fed to finishing cattle.

Gain and efficiency differences with increased inclusion of Rye observed in this experiment are consistent with the dilution of NE caused by the substitution of Rye for DRC. Other researchers have reported that the limited inclusion levels of rye grain (<30%) did not affect the performance of growing Holstein calves (Sharma et al., 1981) or finishing dairy bulls (Huuskonen and Pesonen, 2018). Those researchers were substituting rye for barley, resulting in experimental diets that were nearly isocaloric, reducing the likelihood of observing performance differences.

Net energy dilution alone does not explain the increased ADG observed during the adaptation period and the lack of treatment effects on G:F from day 20 to day 47 in the current experiment. Negative effects of increased Rye inclusion on DMI, growth response, and feed efficiency were not apparent until after day 47. Taken together, these observations support the conclusions that exposure to increased amounts of ingested Rye or exposure over a prolonged period negatively affects growth and efficiency in finishing beef steers.

One explanation for differing DMI and ADG responses over time is ergot alkaloid exposure. Much more research has been conducted on the effects of alkaloids associated with endophyte-infected fescue compared to those produced by *Claviceps pupurea* in ergot-infested grain; however, toxicosis and ergopeptide alkaloids are similar between the two sources (Evans et al., 2004). Dietary ergot alkaloid concentrations were 0, 78, 157, and 235 ppb for 60:0, 40:20, 20:40, and 0:60, respectively, based on total ergot alkaloid concentration in the hybrid cereal rye used in this experiment. (Matthews et al., 2005) observed decreased DMI of endophyte-infested fescue with ergot alkaloid concentration of 120 ppb. Growth and efficiency decreases have been observed with ergot alkaloids as low as 150–200 ppb (Evans et al., 2004; Klotz, 2015), suggesting that longer-term exposure to ergot alkaloids could cause receptor accumulation, leading to larger impacts on biological processes. In this experiment, as days on feed and DMI increased, increased inclusions of Rye could have caused DMI and performance decreases later in the feeding period, particularly in the 20:40 and 0:60 treatments.

Altered cattle feeding behavior could be another explanation for these differing responses over time. This experiment was not designed to quantify changes in feeding patterns; however, it was apparent by the daily observations that pens assigned to treatments with greater inclusion of Rye took more time to consume their daily ration compared to 60:0 (S. Bird, personal communication). This agrees with experiments where steers fed endophyte-infested fescue seeds ate more slowly compared to their pair-fed counterparts on a negative control diet (Ahn et al., 2020). Decreased rate of DMI with increased inclusions of Rye in this experiment could have provided an advantage by mitigating subacute acidosis risk during the early portion of the experiment but negatively affected DMI later in the experiment.

The degree of rye processing also may have contributed to decreased DMI. The PI for Rye chosen for this experiment was based upon suggested PI used for barley and wheat in finishing diets to minimize excess fines while enhancing ruminal starch degradability (Koenig and Beauchemin, 2011). Excessive fines created by increased grain processing can depress DMI (Zinn, 1993). Excessively processing the Rye in this experiment could have caused reduced DMI with increased inclusion rates of Rye. The lack of response during the adaption phase could be explained by an increased amount of roughage fed during this period, mitigating the effects of rapid ruminal starch degradation. Starch from rye grain was more degradable in situ than either barley (Krieg et al., 2017) or corn (Rajtar et al., 2020). In the latter experiment, the degree of processing had less effect on rye compared to corn. Because of differences in starch degradability and response to grain processing, the optimal PI for cereal rye may be less than that of other cereal grains, such as barley or corn.

The DPs for all treatments were less than expected in this experiment but consistent with the results observed by others during winter in the Midwest (Pusillo et al., 1991; Busby and Strohbehn, 2008). The quadratic effect of Rye inclusion on DP could be related to increased concentrations of dietary NDF and ADF; however, increased concentrations of NDF and ADF caused by increased inclusions of dry-rolled barley substituting for DRC had no effect on the DP (Johnson et al., 2020). Feeding endophyte-infested fescue seeds increased the total weight of rumen contents (Ahn et al., 2020), which might explain the DP changes observed in the current experiment as dietary ergot alkaloid intake increased. Conclusions regarding the effects of Rye inclusion on the DP should be made cautiously because of the confounding effects of muddy pen conditions in this experiment.

Indicators of carcass fatness (RF, KPH percentage, RY, and YG) were unaffected by treatment. Longissimus muscle area and AFBW decreased with increased Rye inclusion, suggesting that Rye decreased muscling and frame size at increased inclusion rates. This result was unexpected and not easily explained. Greater substitution of rye grain for DRC decreased the dietary energy density. This, combined with the decreased DMI observed in the current experiment, should have resulted in less fat deposition with increased inclusions of Rye. An MP deficiency is consistent with decreased DMI and AFBW; however, MP supply was greater than the requirements in this experiment (NASEM, 2016). Very little work has been done evaluating the effects of ergot alkaloid contamination in finishing cattle diets; however, it has been suggested that ergot alkaloids can negatively affect energy metabolism independent of DMI (Coufal-Majewski et al., 2016).

## CONCLUSION

These data indicate that Rye can be successfully fed to finishing beef steers. In this experiment, NEm and NEg values of Rye were 87.6% and 83.9% of the values for DRC, respectively, when completely replacing DRC. Blends of two-thirds DRC to one-third Rye supported increased carcass-adjusted growth performance and DMI compared to increased inclusions of Rye. Therefore, the combination of DRC and Rye could be a tool to optimize the NE content of Rye when fed to finishing cattle. Additional work is required to determine if the negative effects of increased rye inclusion on DMI and performance were caused by cereal rye per se, ergot alkaloid concentrations, or the degree of grain processing.
